# Replicability of representational similarity and its role in successful memory retrieval

**DOI:** 10.1162/IMAG.a.1214

**Published:** 2026-04-24

**Authors:** Ece Yuksel, Erica S. Shafer, Madeline Netto, Rachel A. Diana

**Affiliations:** Department of Psychology, Virginia Tech, Blacksburg, VA, United States

**Keywords:** fMRI, representational similarity analysis, episodic memory, hippocampus, context variability, encoding variability

## Abstract

Studies of hippocampal pattern similarity during event encoding and its relationship to subsequent memory retrieval have revealed inconsistent results. Our laboratory recently found evidence that differences in cognitive processing during encoding can modulate the relationship between hippocampal pattern similarity and recognition success. This finding is consistent with the theoretical proposal that hippocampal representations have a dynamic relationship to memory retrieval in which cognitive goals are influential. However, there have been few attempts to replicate representational similarity findings from functional magnetic resonance imaging (fMRI) and, to our knowledge, no evidence of successful replication in either the hippocampus or subsequent memory studies. In order to draw strong theoretical conclusions from our findings, or others in the literature, it is important to demonstrate that those findings are robust. The current study attempted a direct replication of our prior experiment with the exception of minor modifications in the neuroimaging parameters, which were intended to assess the degree to which representational similarity analyses are influenced by reasonable technical differences in data collection. We did not replicate the finding that cognitive variability interacts with future recognition success in the hippocampus. Overall, we failed to replicate 9 out of 12 significant *F*-test results from the Lim et al. study. The three findings that were replicated can be explained by minor visual differences in stimulus presentation on variable cognitive context trials. In addition, we found three new significant effects in the current study that did not previously appear in our earlier study. Therefore, we conclude that fMRI studies using representational similarity analysis of subsequent memory performance are sensitive to minor methodological variation. Further tests of the replicability of these findings are needed prior to drawing theoretical conclusions from their results. Preregistered Stage 1 protocol: https://osf.io/njzhq (date of in-principle acceptance: August 1, 2025) Final recommended Stage 2 manuscript and materials: https://doi.org/10.17605/OSF.IO/ZUYNA PCI:RR Stage 1 recommendation: https://rr.peercommunityin.org/articles/rec?id=873 PCI:RR Stage 2 recommendation: https://rr.peercommunityin.org/articles/rec?id=1278

## 1 Introduction

The episodic memory system encodes information about personally experienced events to allow future retrieval. This information includes slowly changing contextual features such as time, space, and cognitive goals. Researchers have established that a functional medial temporal lobe (MTL) memory system, particularly the hippocampus, is essential for encoding new event memories (e.g., Dickerson & Eichenbaum, 2010; Milner, 1972). These memories are thought to arise from biological coding through neuronal patterns of connection and communication within those brain regions (e.g., Phan et al., 2024; Sugar & Moser, 2019). A central question of interest in the study of episodic memory is how neural codes in the hippocampus are associated with memory performance.

Subsequent memory analyses allow us to study how patterns of neural activity during an event relate to later retrieval of that event from memory (Paller & Wagner, 2002; Wagner et al., 1998). Functional magnetic resonance imaging (fMRI) studies of subsequent memory examine neural patterns by analyzing the blood-oxygen-level-dependent (BOLD) signal in voxels, which typically range from 1 to 3 mm³ in size. Early fMRI studies of subsequent memory were limited to identifying areas where clusters of voxels increase or decrease their activation, as a group, during an event that was later remembered as compared with one that was later forgotten. More recent methods of analyzing fMRI data allow researchers to identify patterns of activity across individual voxels that predict subsequent memory. That is, a collection of voxels in which some increase their activation, others decrease their activation, and still others remain at the same level can be associated with later memory retrieval. The simplest approach to analyzing such activity patterns is known as representational similarity analysis (RSA; Kriegeskorte et al., 2008). While voxel patterns do not capture neuronal-level representations, prior studies show that these patterns are sensitive to a variety of stimulus features (e.g., Dimsdale-Zucker & Ranganath, 2018).

Representational similarity applied to a study of subsequent memory is always a relative comparison. That is, the correlation between voxel patterns of remembered events is compared with the correlation between voxel patterns of forgotten events. However, that overall comparison is typically divided further into groups of events that have information in common, allowing inferences to be made about the specific information contained within event memory representations (Brunec et al., 2020). The current study compares repetition of an item stimulus (defined as the central focus of attention and the information that will later be recognized from memory) across two distinct temporal events. This design allows us to investigate whether the informational content of an event affects its memory representation and whether that factor influences the likelihood of remembering or forgetting that event during a later memory test.

Existing theories of memory representations and existing data provide support for all three possible outcomes of RSA studies: representational similarity among similar events predicting subsequent memory, no relationship between representational similarity and cognitive similarity, and representational dissimilarity among similar events predicting subsequent memory. We briefly review example theories and data for each outcome below in order to demonstrate the variability in the current literature using RSA to investigate subsequent memory.

The idea that similar memory representations should predict successful memory retrieval is featured in several theories. Nadel and Moscovitch’s “multiple trace theory” (1997) provides a useful perspective for thinking about hippocampal representations of episodic memory and the relationships between similar episodic traces. In particular, the ideas that retrieving/reactivating a memory representation always occurs in a new “neuronal and experiential context” (Nadel & Moscovitch, 1997, p. 223) and that such reactivation always results in the creation of a newly encoded hippocampal memory representation are helpful to the theoretical questions being investigated in the current study. This neural theory can then be linked to Hintzman’s (2004) “recursive reminding” hypothesis, which accounted for behavioral findings in judgments of frequency and recognition confidence. Recursive reminding proposes that participants are likely spontaneously recall earlier, related, events during study trials (i.e., reminding). It also proposes that any reminding that occurs during an event is part of the cognitive context of that event, and, therefore, might be part of the neural representation of that event. Although reminding may be more or less likely under different circumstances, repeating items within the same experimental session, as in the current study, is a condition under which reminding is likely. If we apply this cognitive theory to neural representations, we might expect that encoding Item 1/Event 1 (e.g., a picture of a dog presented as trial 14 in a series) produces a particular pattern of neural activity that serves as the memory representation for that experience. Retrieving that item would then involve reinstating a similar pattern of neural activity. In paradigms such as the current study, encoding Item 1/Event 2 (e.g., a repetition of the picture of a dog that is now presented as trial 30 in a series) will sometimes lead the participant to retrieve the Item 1/Event 1 experience and, therefore, to reinstate its memory representation. If so, we might expect that the neural representation of Item 1/Event 1 will sometimes be incorporated into the neural representation of Item 1/Event 2. Therefore, we might hypothesize that reminding during an encoding task will increase the similarity between the memory representations stored for two instances of a single item. Indeed, several studies have found that increased similarity in the hippocampal neural pattern during repetition of an event is related to increased memorability of that event (e.g., Tompary et al., 2016; van den Honert et al., 2016).

However, this intuitive hypothesis that neural similarity reflects cognitive similarity conflicts with computational models and neuroscientific data suggesting that representations in the hippocampus are subject to “pattern separation” (see Yassa & Stark, 2011 for a review). In particular, the dentate gyrus of the hippocampus is thought to convert similar events into orthogonal representations (e.g., Berron et al., 2016; O’Reilly & McClelland, 1994). This separation of representational patterns prevents interference among similar events in episodic memory (and in hippocampal computations). The principle of pattern separation suggests that cognitive similarity among events, like that described in the recursive reminding hypothesis, would not produce a corresponding similarity in the hippocampal representations of those events. Indeed, pattern separation suggests that cognitively similar events are no more similar to or different from one another than cognitively dissimilar events in terms of their neural representation in the hippocampus. Evidence for such a pattern in an RSA dataset would require drawing conclusions from null correlations between hippocampal representations and later memory performance, which is not generally encouraged in the current literature. Therefore, it is difficult to determine how often such a pattern has been identified. However, there is at least some evidence that hippocampal patterns do not always carry information about memorability of a stimulus even when medial temporal lobe cortical regions do so (e.g., Martin et al., 2013).

A third possible pattern for hippocampal representations is that similar events become more differentiated from one another than dissimilar events. This pattern has sometimes been attributed to gradual learning across a task that requires participants to discriminate between highly similar stimuli (Favila et al., 2016; Hulbert & Norman, 2015). However, other studies that do not clearly encourage discrimination between similar objects have also found an association between differentiation and memory retrieval in the hippocampus. For example, a study that required participants to monitor the color of a fixation cross prior to a surprise memory test found hippocampal differentiation (LaRocque et al., 2013). A different study that used distinct object pictures and tested temporal order judgments also found hippocampal differentiation (Jenkins & Ranganath, 2016). Therefore, it is not yet clear under what circumstances differentiation will support memory rather than orthogonal representations or similar representations.

Indeed, a recent review of studies that report hippocampal pattern analyses highlighted the complexity of this literature and the inconsistency of findings (Brunec et al., 2020). The literature is complex because studies use a wide variety of stimuli, encoding procedures, retrieval paradigms, and analysis approaches. For example, some studies report hippocampal pattern similarity as a function of subsequent memory performance (as in the current study), whereas others report hippocampal pattern similarity either in relationship to other behavioral measures (e.g., vividness) or without relationship to behavior. Some studies examine pattern similarity within repeated events, whereas others examine pattern similarity across a range of stimuli that have common features (e.g., a category of object). When this challenging body of evidence was distilled, Brunec et al. concluded that hippocampal representations can either integrate or differentiate events, depending on the task goal. They specifically recommend that “future studies should test whether increases and decreases in similarity can both facilitate memory depending on different experimental parameters, such as stimulus characteristics, task demands, time of testing and precise hippocampal localization…this line of research would provide some insight into perhaps one of the most critical issues regarding this field of study: how the same realworld experience is represented depending on whether we are attempting to extract generalities or to draw on specific experiences” (Brunec et al., 2020, p. 205).

Our laboratory recently published a study (Lim et al., 2023) that specifically investigated the role of task instructions/encoding context in modulating the relationship between hippocampal representations and subsequent memory retrieval. We presented pictures of distinct objects and asked participants to make behavioral judgments in response to two semantic processing questions. Each question emphasized different features of an object (usefulness/function vs. shape/size/weight). The key manipulation was in the repetition of each object image. For half of the items, the picture was presented twice with an identical processing question and, therefore, the participant was likely to consider the same features during both events (“Same Context”). For the remaining items, the picture was presented with a different processing question during its repetition and, therefore, the participant was likely to consider distinct features during the two events (“Variable Context”). Our research has indicated that increased variability in cognitive processing of this type leads to improvements in recognition memory performance (Lim et al., 2023; Salan et al., 2024).

When we tested an fMRI measure of representational similarity between object repetitions, we found that hippocampal representations differentially predicted subsequent memory depending on the consistency of the cognitive tasks applied during encoding. That is, correlations among the voxel patterns in our hippocampal region of interest on the first and second presentation of an identical object image showed a significant interaction effect between contextual variability (Same Context vs. Variable Context) and subsequent memory retrieval (Remembered vs. Forgotten). This pattern was not found in the other three regions of interest that we investigated: parahippocampal cortex, perirhinal cortex, or lateral occipital cortex. Although we had predicted this pattern *a priori*, our prediction was for the medial temporal lobe cortex (parahippocampal cortex and perirhinal cortex) rather than the hippocampus. We interpreted this finding as indicating support for the conclusion made by Brunec et al. (2020) that hippocampal representations for related memories have different relationships to one another, depending on cognitive goals. As noted by Brunec and colleagues, this conclusion would help to explain the inconsistent findings within the literature relating representational similarity to subsequent memory and suggests a path forward for future studies.

However, this progress is hampered by minimal evidence of replicability in representational similarity studies of subsequent episodic memory. If variability in the current literature is to be interpreted as indicating adaptive hippocampal function, we must be sure that it is possible to replicate findings when the conditions are held constant. As of August 2024, a PubMed search with the terms “representational similarity analysis” AND fmri AND (replicability OR replicate OR replication) produced 20 results. Of those results, 12 studies use the term replication to refer to other types of analyses, for example, replication of a behavioral finding, and will not be examined further.

Of the eight studies that reported attempts to replicate RSA findings, one study reported findings that did not replicate across development, comparing a group of children with a group of adults (Mitchell et al., 2021). The authors interpreted this finding as indicating that development changes the nature of emotional processing. A further two studies (Charest et al., 2018; Schone et al., 2021) replicated RSA findings within a single dataset by using multiple analysis approaches to reach the same conclusion. Although this provides some evidence that RSA conclusions are robust to reasonable analytical variation, it does not indicate that RSA conclusions are robust across participant groups or across neuroimaging sequence changes.

This leaves five instances in the current literature that report some degree of replication of RSA findings in separate datasets. Three of these studies investigate semantic similarity in regions including posterior middle/inferior temporal gyrus and precuneus (Liuzzi et al., 2020), intraparietal sulcus (Neyens et al., 2017), and perirhinal cortex (Bruffaerts et al., 2013). None of these studies reported findings in the hippocampus. A fourth study (Mohsenzadeh et al., 2019) examined visual perception at the whole brain level using a combination of fMRI and magnetoencephalography (MEG) data. This study focused on the hierarchy of visual processing from primary visual cortex through the dorsal and ventral streams and did not report any findings in the hippocampus.

The last of the five RSA replication studies is the only one that could be considered related to episodic memory and measures of subsequent retrieval and it is also the only replication study that reported findings in the hippocampus (Visser et al., 2015). Visser and colleagues used a fear conditioning paradigm to replicate their prior finding (Visser et al., 2013) that representational similarity during encoding predicts later pupil dilation measures of fear responses. The authors describe this paradigm as “long-term procedural fear memory” rather than explicit, conscious, episodic memory. As we describe below, the evidence for replication of the initial findings is somewhat weak and does not occur within the hippocampus.

The analysis of subsequent fear responses in the earlier study (Visser et al., 2013) divided participants into separate groups according to their pupil responses to images previously associated with shock (retention vs. no retention groups). The participants who showed larger pupil size differences to the shock-associated stimulus than to the non-reinforced stimulus during the post-test (the retention group) also had more differentiated patterns (i.e., negative correlations between stimuli) in the hippocampus during initial fear learning than participants whose pupil size showed the opposite pattern (Visser et al., 2013). The replication study (Visser et al., 2015) uses a different analysis technique because only 3 of the 39 participants showed the “no retention” pupil response pattern in the new study. However, the Supplementary Materials in Visser et al. (2015) indicate that the hippocampal correlation for between-stimulus comparisons was not associated with pupil dilation response (r = 0.047, *p* = 0.776).

None of the within-stimulus pattern similarity measures were associated with subsequent implicit fear responses (Visser et al., 2013). Instead, a test of within-stimulus pattern similarity in the hippocampus (as proposed for the current study) demonstrated higher similarity for stimuli that were later remembered in an explicit memory test (correct identification of the shock-associated and sound-associated face and house stimuli) than for those that were later forgotten. However, no hippocampal effects for explicit memory were reported in the replication manuscript, likely because 36 out of 41 participants correctly retrieved all associations. Therefore, there was no replication of the hippocampal effects for explicit associative memory (Visser et al., 2015).

Instead, the replicated RSA findings were areas outside of the hippocampus. The later study (Visser et al., 2015) reports replicating the association of between-stimulus pattern similarity during the learning session with pupil dilation during the test session in the anterior cingulate cortex, insula, and ventromedial prefrontal cortex. However, it also notes that the insula finding was not significant when uncorrected (*p* = 0.064) and the remaining findings were not significant after correction for multiple comparisons. When considering that only a subset of regions of interest showed evidence of replicated effects and that those regions did not survive the intended statistical corrections, we interpret these studies as providing only weak evidence of replication and none in the hippocampus.

Several changes were made across the studies that might account for the differences in findings. The authors favor the conclusion that shortening the retention time between fear conditioning and pupil dilation measures in the later study led to ceiling effects. Relevant to the current investigation, the authors also changed some of the neuroimaging parameters from the first to second study. The earlier study (Visser et al., 2013) used an axial slice orientation with 37 slices and ascending acquisition order, whereas the later study used a sagittal slice orientation with 39 slices collected using interleaved acquisition (Visser et al., 2015). Both studies used the same scanner, head coil, voxel sizes, repetition time, echo time, and flip angle. Although retention time and ceiling effects might be expected to have an effect on the outcome of a memory study, it is important to establish that minor differences in imaging procedures do not change the conceptual outcome of such studies. RSA studies are intended to identify pattern similarity while remaining agnostic to reasonable variations in fMRI scanning parameters.

The successful RSA replications published in the literature and reviewed above provide further insight into this possibility. One of the successful replications (Bruffaerts et al., 2013) was a within-manuscript replication that used identical neuroimaging parameters for both datasets. Among the other three replication studies, the following parameters were varied across only one of the sets that produced a clear replication: scanner strength and head coil (4 Tesla, 8-channel head coil, Fairhall & Caramazza, 2013; 3 Tesla, 64-channel head coil, Liuzzi et al., 2020), repetition time/TR (750 ms, Cichy et al., 2016; 2000 ms, Mohsenzadeh et al., 2019), and voxel size (3-mm isotropic, Devereux et al., 2013; 2.75 mm x 2.75 mm x 3.75 mm, Neyens et al., 2017). Other parameters were varied across two of the three sets that produced a clear replication, indicating that the findings were fairly robust to these changes: flip angle, echo time/TE, and acceleration factors. Although not all studies explicitly reported the slice orientation used, it does not appear that any of the three successful replications varied the slice orientation from one study to another. Therefore, we selected slice orientation as the most important methodological factor to vary in the current replication attempt. We also elected to vary voxel size because it is both methodologically important in determining the specific BOLD pattern that is measured and conceptually irrelevant to the conclusions that an RSA study is meant to draw.

Our review of the existing literature leads us to conclude that there have been very few successful replications of RSA studies. Within those few replications, we did not find any studies that replicated either subsequent memory effects in explicit memory or hippocampal findings. Whether this indicates that such studies are rarely attempted or are rarely successful cannot be determined and, therefore, a registered replication attempt is clearly needed. Therefore, we proposed to replicate our recent study (Lim et al., 2023) with altered imaging parameters in order to assess whether this finding, and representational similarity analyses of subsequent memory as currently performed, can be relied upon for theoretical conclusions. The goal of this study was to hold constant all factors that have conceptual relevance to the interpretation of the results while allowing some of the tangential methodological factors to vary. Therefore, finding the same interaction between the cognitive encoding manipulation and memory retrieval in the hippocampus would indicate support for Brunec’s proposal that variability in the fMRI literature with respect to hippocampal pattern similarity and subsequent memory is driven by identifiable cognitive factors. However, failing to find this interaction would indicate that this same variability in the literature may be driven by methodological inconsistencies and will call attention to a need for further refinement of representational similarity analysis techniques in fMRI studies of memory.

## 2 Methods

### 2.1 Participants

A power analysis (G*Power 3.1.9.2) indicated that 95% power to detect an effect size of 0.44 in an *F*-test repeated measures interaction requires a minimum sample size of 26. This power analysis was based on the key interaction between context variability and memory retrieval in the hippocampal region of interest (ROI), from study we aim to replicate (Lim et al., 2023). However, a second key finding in that study suggests that a larger sample size is necessary, with 95% power to detect an effect size of 0.51 in a paired-samples *t*-test requiring a minimum sample size of 43. This second power analysis was based on the key *t*-test in that same study, which showed that encoding of correctly remembered items in the Variable Context condition produced lower pattern similarity than did encoding of forgotten items in the hippocampus. Therefore, we recruited participants for this study until we had 43 usable datasets, replacing any participants who did not meet our inclusion criteria. Inclusion criteria for differentiating usable from unusable data were originally registered as overall memory performance of *d’* > 0.75, successful manipulation check performance (described in section 2.3), and a minimum of 20 trials per condition after removing movement outliers and items for which no behavioral response was collected during encoding. Movement outlier trials were identified via the Artifact Detection Tools (RRID:SCR_005994) plugin in SPM. All inclusion decisions were made prior to calculation of RSA correlations for any participant. The Lim study’s (2023) dataset had an exclusion rate of 35% without any manipulation check criteria. The Salan study’s (2024) dataset for Experiment 4, which did exclude participants for manipulation check performance, had an exclusion rate of 23% (including 20 participants, out of 101, who were excluded due to manipulation check performance and 3 who were excluded due to memory accuracy). Based on these numbers, we expected to collect a total of 56 to 66 participants in order to meet our minimum included sample size of 43. Mid-way through data collection, the number of excluded participants was higher than anticipated. Therefore, we requested and were approved to register a deviation from the planned inclusion criteria. This deviation was registered before any RSA data were examined. We lowered the overall memory performance criterion to *d’* > 0.50, successful manipulation check performance (described in section 2.3), and a minimum of 19 trials per condition after removing movement outliers and items for which no behavioral response is collected during encoding.

As in our prior study (Lim et al., 2023), handedness was measured via a simplified version of the Edinburgh Handedness Inventory (Oldfield, 1971). In order to recreate the characteristics of that earlier dataset, which ultimately included 3 left-handed participants and 27 right-handed participants, we limited the number of left-handed participants in the current study to 4 or fewer (10% of the total N).

Participants were limited to those 18 to 50 years of age, as in the prior study, and those who were medically eligible for MRI scanning. The study was approved by the Human Research Protection program at Virginia Tech via external review from BRANY (Biomedical Research Institute), file # VT18-1077-568(TRX). Informed consent was obtained from all participants for being included in the study.

The final included N was 43 participants (description of total number collected and applied inclusion criteria are given below). One participant had no remaining voxels in left perirhinal cortex due to signal dropout and movement. Therefore, analyses for the perirhinal cortex region of interest include only 42 participants. Participant ages ranged from 18 to 35 years, with a mean of 25.74 years and a standard deviation of 3.84. The gender distribution of these participants was 19 male participants, 21 female participants, 2 nonbinary participants, and 1 participant who preferred not to report gender. The self-reported racial and ethnic identities of these participants included 17 White, 11 Asian, 5 South Asian, 2 White/Hispanic, 2 Black/African American, 1 African, and 5 reporting multiple categories (2 Asian/White, 2 Black/White, and 1 Middle Eastern/White. The handedness inventory indicated that 4 participants were primarily left-handed (using the left hand to write) and 39 participants were primarily right-handed.

The raw dataset included 63 participants, of whom 5 did not complete the required study and test phase trials. Of the 58 participants who completed the task, 10 were excluded due to insufficient trial numbers after skipped study responses and movement outliers were removed, 2 were excluded due to overall *d′* below 0.50, and 3 were excluded for failing to meet multiple inclusion criteria (a combination of manipulation check response errors and either insufficient trial numbers, insufficient accuracy, or both). All inclusion/exclusion decisions were made prior to inspecting any RSA correlations. If we had used the originally planned exclusion criteria (identical to those in the Lim et al. study), 12 would have been excluded due to insufficient trial numbers, 7 due to overall *d′* below 0.75, and 3 due to multiple reasons (for a final N of 36).

### 2.2 Materials

Image stimuli were identical to those used in our prior study (Lim et al., 2023), which were selected from the Bank of Standardized Stimuli (Brodeur et al., 2010). A total of 416 object images were used, with half randomly selected as study items for each participant and the other half used as unstudied items during the memory test. We used the same semantic encoding questions as in our prior study to differentiate contexts. Both questions ask participants to think about the item in the image but they also direct the participant to attend to distinct characteristics of the item and to place the item in a distinct environment. In order to encourage the participants to think carefully about each question, we began each context block with self-paced presentation of longer, more detailed versions of the questions and accompanying rating scales. The deserted island question was given as “Imagine that you were stranded on a deserted island with no other people and only the natural resources of the island to help you survive. In that scenario, if you were to find the object shown in the picture on the island would that object be useful to help you survive?” The shortened version of the deserted island question shown on each trial was “Is this item useful on a deserted island?” with the rating scale choices labeled “Not useful”, “Somewhat useful”, and “Essential.” The item carrying question was given as “Imagine that there is an emergency that requires someone to carry the item shown in the picture across a large field to the other side. You could carry it on your back, in your arms or in a backpack if that is helpful. If you were the only person available to do it, would you be able to carry the object?” The shortened version of the item carrying question shown on each trial was “Could you carry this item a long distance?” with the rating scale choices labeled “Impossible”, “Possible but hard”, and “Very easy.”

### 2.3 Procedure

The procedure was identical to our prior study (Lim et al., 2023) with four exceptions: addition of behavioral manipulation check items, slightly shortening the intertrial interval, changing the slice orientation, and reducing the voxel size. Each of those changes and the motivations for the changes are explained in the relevant section of the procedure below.

As in our prior study, the encoding trials were shown during fMRI scanning with the retrieval task scheduled between 8 and 10 days later outside of the scanner. This delay between encoding and retrieval led to approximately equal numbers of items forgotten and remembered, allowing for robust analysis of subsequent memory performance. The delay between study and test for included participants averaged 208 h (8.66 days), with a standard deviation of 20 h (0.82 days), a minimum delay of 181 h (7.52 days), and a maximum delay of 241 h (10.03 days).

During encoding, each trial consisted of an object image presented with one of the two semantic processing questions described in section 2.2 above. Each image was presented twice during encoding with the repetition context being manipulated. Context was defined as the continuous block of trials within which the same semantic processing question was shown on all trials. Average repetition spacing was controlled across conditions and condition order was counterbalanced across participants.

The independent variable in our behavioral task was context variability, defined according to the semantic processing question or questions that were applied during the first and second presentation of each object image. This variable has two levels: Same Context and Variable Context. Items in the Variable Context condition were studied once with each of the two semantic processing questions. Items in the Same Context condition were studied twice with an identical semantic processing question. The two semantic processing questions occurred equally often in both conditions.

Our fMRI analyses include a second pseudo-independent variable: subsequent memory retrieval, which is defined according to the memory response given to each item during the delayed memory test. This variable also has two levels: subsequently remembered items (old items correctly identified as old) and subsequently forgotten items (old items incorrectly identified as new).

A custom MATLAB script was used to randomize the images used as studied items, their assignment to the Same or Variable Context conditions, and position on the study list for each participant. Using this script, repetition spacing for both the Same and Variable Context conditions was set between 8 trials and 16 trials for each image, with an average of 12 intervening trials between an image’s first and second presentation. As in our prior study (Lim et al., 2023), spacing could not be held constant without repeating images in the identical order, which might encourage participants to encode relationships across stimuli. Table 1 shows the mean trial lag between repetitions of the first and second presentation for each object in the Same Context and Variable Context conditions, divided by hit and miss recognition responses, for the 43 included participants.

**Table 1.** Mean number of trials between first and second presentations of object images in the Same Context and Variable Context conditions, divided by subsequent correct old responses (hits) vs. incorrect new responses (misses).

	Same context	Variable context
Hit	12.50 (0.64)	12.50 (0.46)
Miss	12.41 (0.55)	12.55 (0.42)

Standard deviations are shown in parentheses.

Participants were given the same instructions as in our prior study: that they should think about each object image as it appears and enter their response to the encoding question on each trial, even if the image has been seen before. Participants were not told that they would be asked to remember the items for a later memory test. A series of practice trials were presented prior to the encoding phase.

We added a behavioral manipulation check to this replication study in order to strengthen the study beyond the prior version. Although the answers to the semantic encoding questions are largely subjective, there are stimuli for which most responders would agree on the answer. In a prior behavioral replication study (see Experiments 3 and 4 in Salan et al., 2024), we found that the addition of manipulation check items that have objectively correct answers to these encoding questions allowed us to identify participants who were not fully cooperating with the encoding instructions, even when those participants’ overall memory performance was sufficient to be included in our dataset. In our behavioral replication, removing participants who did not meet an *a priori* exclusion criteria based purely on these manipulation check items led to a substantial increase in the effect size. This led us to conclude that such manipulation checks are important in studies where the key manipulation is dependent on the participants’ cooperation. Given the importance of the behavioral manipulation for our fMRI findings, we expected that a similar manipulation check would increase the quality of our dataset. Therefore, as in Experiment 4 of our prior behavioral study (Salan et al., 2024), we included manipulation check objects for each of the two encoding questions, pseudo-randomly distributed throughout the encoding trials. Two objects for each question had a clear “yes” answer (e.g., campfire for “If you were stranded on a deserted island, would this item be useful?) and two objects for each question had a clear “no” answer (e.g., volcano for “Could you carry this item a long distance?”). All eight of these manipulation check items were repeated once, allowing us to assess both the objective correctness of the encoding responses and the consistency of the response. The objects used on the manipulation check trials were not tested during the retrieval phase nor were they analyzed for representational similarity. Participant responses on those 16 encoding trials were examined prior to calculation of RSA correlations. We excluded participants from the dataset for any of the following 3 criteria: skipping 2 or more responses (out of 16), responding inconsistently across repetition of 2 or more objects (out of 8 total), or making 7 or more individual responses that we deemed incorrect.

**Fig. 1.**
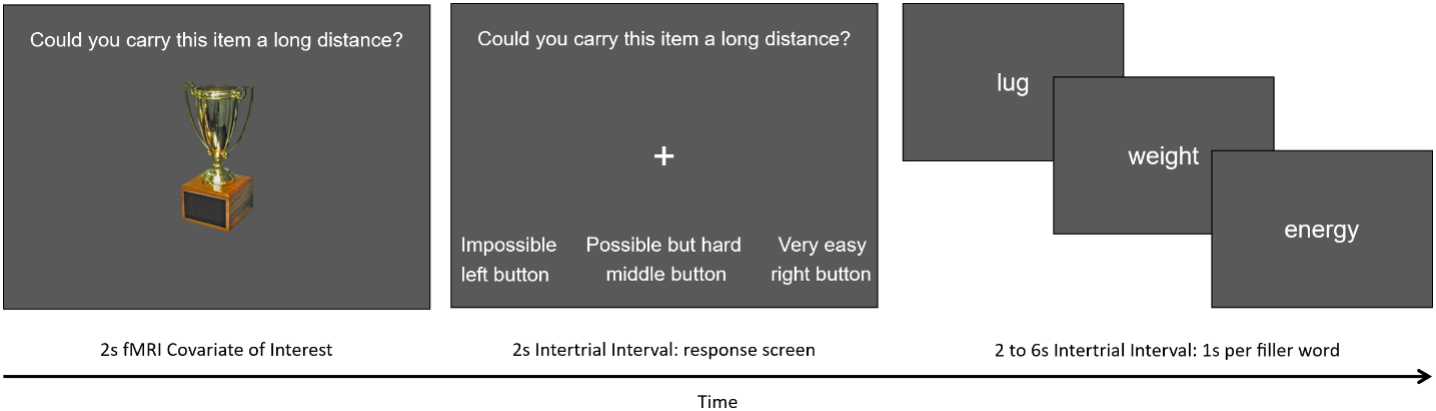
Schematic representation of an encoding trial from the MRI scanning session.

The same experiment script was used as in the prior study (Lim et al., 2023), created with *Presentation* software (Version 18.0, Neurobehavioral Systems, Inc., Berkeley, CA). The onset of each encoding trial was synchronized to a scanner pulse. Figure 1 is a schematic representation of an encoding trial. Each trial consisted of an object image and the short version of an encoding question, shown for 2 s. Immediately afterward a fixation cross appeared with the question and coordinating response scale for 2 s. Participants were asked to make their button press response during this interval. Next, during the intertrial interval, filler words were presented that were related to the encoding question used on each block, one per second. The filler task was designed to engage participants’ attention and discourage intentional rehearsal of the studied items. Participants were told to read the words while they waited for the next image. The unscanned memory test consisted of 208 studied images and 208 new images, presented individually in random order. Participants were asked to respond “Yes/Old”, with the “f” key, or “No/New”, with the “j” key, to the question “Have you studied this image?”. Each image appeared on the screen until the participant responded.

Our prior study (Lim et al., 2023) used an intertrial interval (ITI) that averaged 6 s (including the 2 s response interval), with a minimum of 4 s and a maximum of 10 s on each trial. As described in our prior study, we chose this ITI based on a review of prior methodological studies (Abdulrahman & Henson, 2016; Mumford et al., 2012). Given that those prior studies indicated an optimal range of 2 to 10 s for our planned analysis and that the specific nature of the ITI is not expected to affect our findings, we tested the robustness of our findings to minor methodological changes by slightly decreasing the ITI in the current study. The ITI, including the 2 s response interval on each trial, was either 4, 6, or 8 s, with an overall average of 5 s. This shorter average ITI also compensated for the addition of the manipulation check items so that the length of the functional scans remained similar to the prior study.

As described in our prior study, all RSA correlations were calculated from within-run repeated trials using within-trial-type similarity comparisons (Dimsdale-Zucker & Ranganath, 2018; Mumford et al., 2014). Although the trials in the current version of the study were 1 s shorter on average, our analysis of temporal autocorrelation in the prior study indicated that temporally based correlation ran 20 TRs before and after each trial. Therefore, a minimum of 8 trials of separation between repetitions with 3.5 TRs per trial in the current study created a minimum of 28 TRs between correlated trials, which minimized the role of temporal autocorrelation in the current study.

MRI data were collected on a 3T Siemens Trio scanner using a 12-channel phased array head coil at the Virginia Tech Corporate Research Center Human Neuroimaging Lab. The functional scan parameters were slightly modified from our prior study in order to test the robustness of our findings to minor methodological changes. In the current study, the gradient echoplanar imaging (EPI) sequence had the same repetition time/TR of 2000 ms. The other parameters changed from the prior study were slice orientation = axial, voxel size = 2.7 mm^3^ isotropic, 32 slices, distance factor of 0, echo time/TE = 30 ms, field of view = 192 mm ^1^. This sequence did not allow for full-brain coverage because decreasing the voxel size necessitated decreasing the number of slices collected. We, therefore, prioritized medial temporal lobe coverage. ROIs for primary analysis were parahippocampal cortex, perirhinal cortex, and the hippocampus, defined using the same markers as our prior study (see Buffalo et al., 2006; Diana et al., 2008). We were also able to recreate the lateral occipital cortex ROI examined in our prior study (Lim et al., 2023). High-resolution structural images were T-1 weighted from an MPRAGE sequence with field of view = 243 mm, voxel size = 0.9 mm isotropic, number of slices = 208.

### 2.4 Analysis plan

Our detailed study design plan is found in Table 2. We used the same analysis procedures as in our prior study. Our primary behavioral measure of interest was proportion hit responses (old items correctly identified as old); however, the *d′* measure of overall accuracy was used to exclude participants with poor recognition performance (*d′* below 0.50).

**Table 2.** Study design table created (with the exception of the Results column) prior to data collection.

Question	Hypothesis	Sampling plan	Analysis Plan	Rationale for deciding the sensitivity of the test for confirming or disconfirming the hypothesis	Interpretation given different outcomes	Theory that could be shown wrong by the outcomes	RESULTS
H1a. Does cognitive variability during encoding drive an interaction between hippocampal pattern similarity and subsequent memory retrieval?	H1a. The factors of Context Variability and Memory Retrieval will produce a significant interaction in the hippocampal ROI. This interaction may or may not also interact with the effect of hemisphere.	H1a. A power analysis was conducted to determine that a minimum sample size of 26 would detect an effect size of 0.44 with 95% power in a repeated measures ANOVA interaction.	H1a. Fisher-transformed correlations between voxel patterns in the hippocampus will be subjected to a 2 x 2 x 2 (context condition, memory performance, and hemisphere) repeated measures ANOVA. A two-way interaction between context condition and memory performance or a three-way interaction (with the third factor of hemisphere) would confirm the hypothesis.	H1a. The effect size used in the power analysis was taken from the hippocampal ROI context variability x memory retrieval ANOVA interaction term in our prior study (Lim et al., 2023) that we are attempting to replicate in the proposed study.	H1a. Finding the specified interaction supports the conclusion that hippocampal representations have a dynamic relationship to memory success that can be influenced by cognitive goals. Failure to find the specified interaction suggests that RSA is sensitive to minor methodological changes and, therefore, may not be sufficiently robust.	H1a. Brunec et al’s theory that RSA findings in the hippocampus show substantial variability due to cognitive factors rather than due to methodological inconsistency And Lim et al.’s interpretation that cognitive variability as an encoding goal leads to beneficial variability in hippocampal patterns.	H1a. Neither the two-way interaction between context condition and memory performance, *F*(1, 42) = 0.72, *p* = 0.40) nor the three-way interaction including the factor of hemisphere, *F*(1, 42) = 0.32, *p* = 0.58, were significant. Therefore, this hypothesis was not confirmed.
H1b. Does cognitive variability during encoding drive an interaction between hippocampal pattern similarity and subsequent memory retrieval with a specific pattern in the Variable Context condition?	H1b. “Variable Context” items will be more dissimilar to one another across repetitions in the hippocampal ROI and that dissimilarity will predict correct recognition.	H1b. A power analysis was conducted to determine that a minimum sample size of 43 would detect an effect size of 0.51 with 95% power in a paired-samples *t*-test.	H1b. A paired samples *t*-test will be used to compare the correlations between repetitions of subsequently remembered items and subsequently forgotten items in the Variable Context condition. This *t*-test investigates the specific pattern in any interaction that might be found in H1a above.	H1b. The effect size used in the power analysis was taken from the hippocampal ROI remembered vs. forgotten *t*-test within the Variable Context condition in our prior study (Lim et al., 2023) that we are attempting to replicate in the proposed study.	H1b. Finding the specified pattern within the Variable Context condition replicates the precise pattern of the interaction in the Lim study, further supporting the conclusion that variable encoding goals lead to a benefit in memory for variable representations.	H1b. Lim et al.’s interpretation that cognitive variability as an encoding goal leads to beneficial variability in hippocampal patterns	H1b. The paired samples *t*-test comparing subsequently remembered items with subsequently forgotten items in the Variable Context condition was not significant: *t*(42) = 0.35, *p* = 0.72. Therefore, this hypothesis was not confirmed

SPM12 was used for pre-processing and for the first-level generalized linear model (GLM) of the fMRI data. Data were co-registered for motion correction, slice time corrected, and realigned via linear registration to each participant’s high-resolution structural MRI. Minimal smoothing was applied (2 mm full-width at half maximum smoothing kernel) as recommended to increase signal to noise (Dimsdale-Zucker & Ranganath, 2018; Op de Beeck, 2010). Each fMRI trial was modeled as the single TR during which the study image was visible. We used a Least-Squares Separate (LSS) model with separate covariates of no interest for each of the four condition types (LSS-4), as recommended by methodological studies (Abdulrahman & Henson, 2016; Mumford et al., 2012). We also included covariates of no interest for head motion, derived from the ART toolbox in SPM (Artifact Detection Tools, RRID:SCR_005994). We implemented the GLM option “FAST” for pre-whitening as recommended to minimize temporal autocorrelation (Corbin et al., 2018; Olszowy et al., 2019).

A vector of voxel values from each region of interest was extracted from the first-level GLMs using custom MATLAB scripts. That vector was compared with the vector representing the same image during its repetition via Pearson correlation. Those correlation values were then Fisher transformed and averaged across the trials within a condition for each participant. We assessed those condition-level correlations across participants using separate 2 x 2 x 2 (encoding context condition by hemisphere by subsequent memory outcome) repeated measures ANOVAs for each ROI. Four anatomical ROIs were examined: the hippocampus, perirhinal cortex, parahippocampal cortex, and lateral occipital cortex (LOC). The medial temporal lobe ROIs were defined using pre-established structural markers (see Buffalo et al., 2006; Diana et al., 2008). The LOC was defined using the same common mask from the Lim et al. (2023) study, which was warped to fit each individual participant’s brain space.

Our two primary hypotheses applied to the hippocampal ROI. We expected to find either a two-way interaction (factors: context variability and memory retrieval) or a three-way interaction (factors: context variability, memory retrieval, and hemisphere) in the hippocampal ROI. We expected to find that the underlying pattern of that interaction included a significant paired-samples *t*-test comparing subsequently remembered items to subsequently forgotten items in the Variable Context condition such that remembered items produced a smaller across-repetition correlation between voxel patterns than do forgotten items. Finding both of these effects would be interpreted as supporting the conclusions from our prior study. In contrast, if we did not find a significant interaction between context variability and subsequent memory or if we found a significant interaction between context variability and subsequent memory that is not driven by the expected pattern in the Variable Context condition (more variability in remembered hippocampal patterns than in forgotten), this would be interpreted as a failure to replicate our findings. Therefore, we would conclude that methodological inconsistency or other as-yet-unknown factors drive variability in hippocampal RSA findings in the literature.

All raw data, custom scripts, RSA output, and statistical output are available at: https://doi.org/10.17605/OSF.IO/ZUYNA.

## 3 Results

### 3.1 Behavioral results

**Fig. 2.**
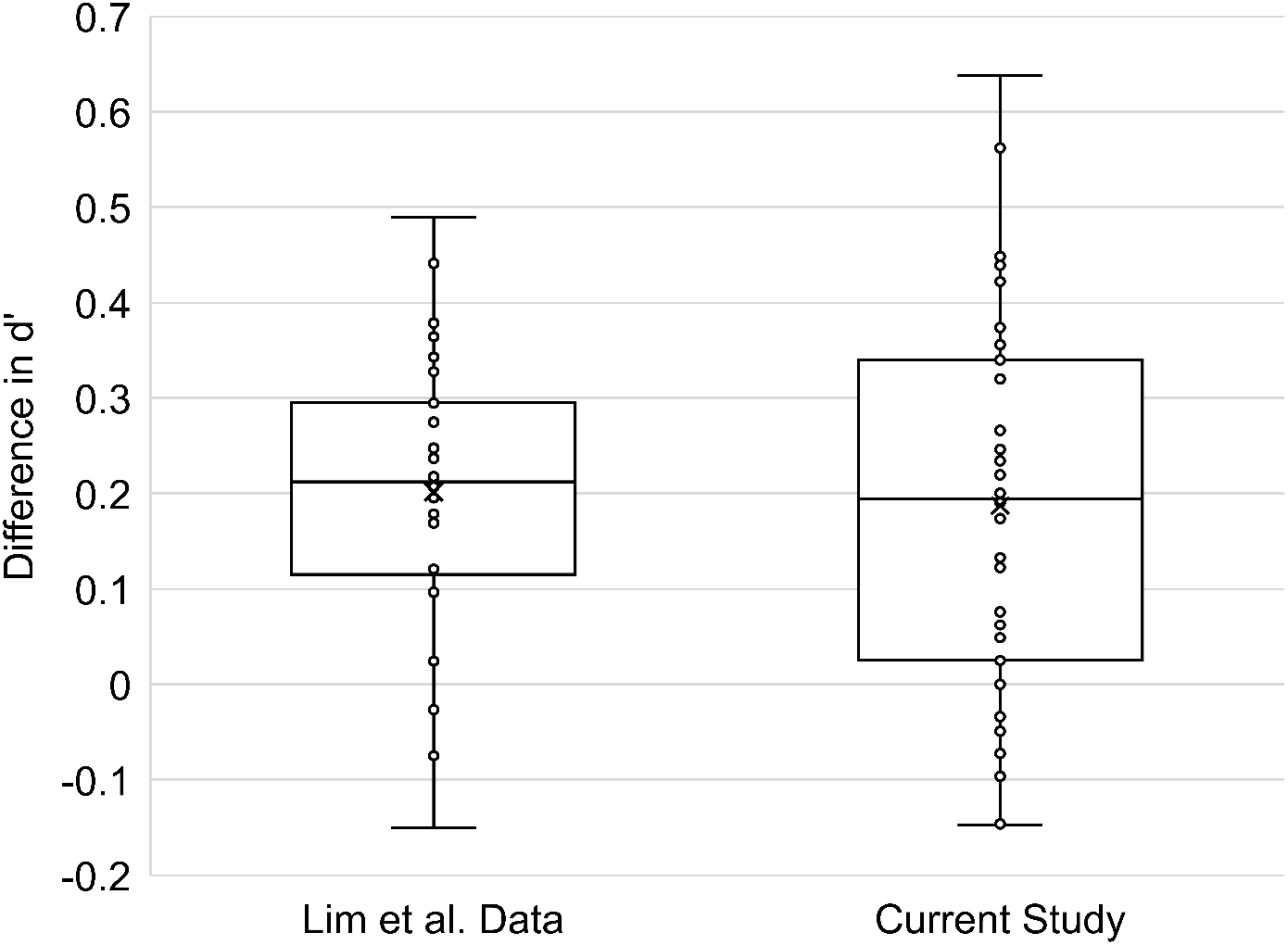
The difference in *d’* between the Variable Context and Same Context conditions for the Lim et al. (2023) study as compared with the current study. Positive numbers indicate higher accuracy for the Variable Context condition than for the Same Context condition. The “X” indicates the mean of each dataset and the horizontal line within each box indicates the median of that dataset. The center two quartiles of the data are presented as the box and the lower and upper quartiles are presented as the lower and upper whiskers. Each circle indicates an individual participant’s *d’* difference.

The current study replicated the behavioral difference between hits in the Same and Variable Context conditions that we have previously found in several studies (Lim et al., 2023; Salan et al., 2024). The Same Context condition produced a mean hit rate of 48.38% (standard deviation = 15.14%) whereas the Variable Context condition produced a mean hit rate of 55.32% (standard deviation = 14.16%). This difference was statistically significant: *t*(42) = 6.40, *p* < 0.001. False alarm rates were low, with a mean of 12.27% (standard deviation = 7.64%). As the false alarm rate did not differ across conditions, the *d’* measure of overall accuracy showed the same pattern as the hit rates (Same Context *M* = 1.21, Variable Context *M* = 1.30). This indicates that the hit rates are reflective of successful memory discrimination relative to false alarms. The difference in *d’* between the Same Context and Variable Context conditions is shown in Figure 2 in comparison with the Lim et al. dataset. Both studies showed the same pattern of behavioral findings, with a consistent memory advantage for the Variable Context condition.

### 3.2 Primary RSA findings

#### 3.2.1 Hippocampal ROI results

**Fig. 3.**
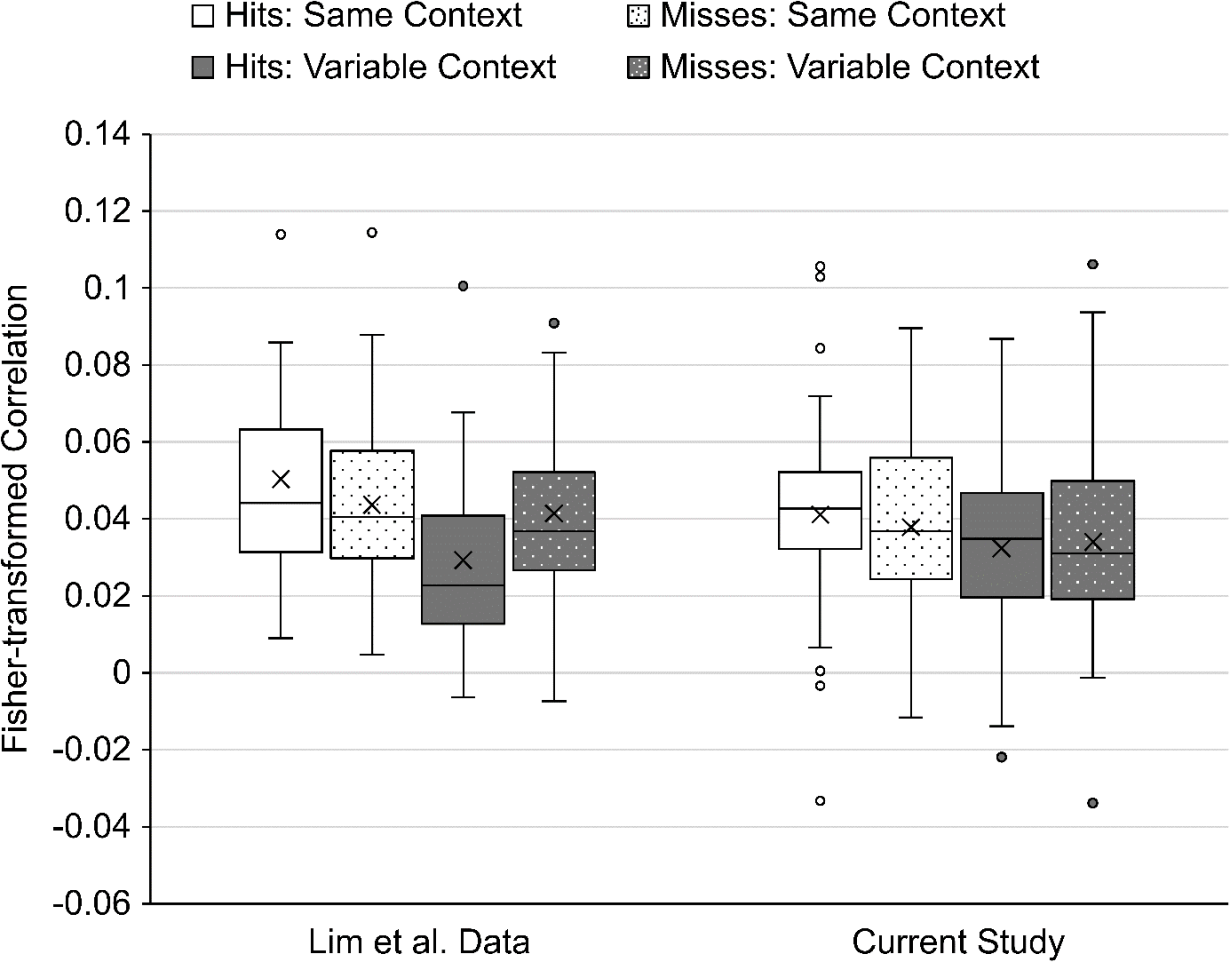
Mean Fisher-transformed correlations for the bilateral hippocampal ROI in the Lim et al. (2023) dataset and the current study. The “X” indicates the mean of each condition and the horizontal line within each box indicates the median of that condition. The center two quartiles of each condition are presented as the box and the lower and upper quartiles are presented as the lower and upper whiskers. Circles indicate individual participants whose correlation value was more than 1.5 times the interquartile range away from the middle two quartiles.

The primary finding of interest in the Lim et al. (2023) study was the interaction in RSA correlations between context variability and memory retrieval in the bilateral hippocampal mask. The current study did not replicate this finding, as seen in Figure 3. In a 2 (context variability) x 2 (memory retrieval) x 2 (hemisphere) repeated measures ANOVA of the findings for the hippocampal ROI, there were no significant main effects or interactions for the RSA correlations (see Table 3). This includes the primary interaction from the Lim et al. (2023) study between context variability and memory retrieval. In the current dataset, the same numerical pattern observed in the previous study was evident (Same Context Hit vs. Miss difference *M* = 0.003, Variable Context Hit vs. Miss difference *M* = -0.002), but the differences were substantially smaller (Lim et al., 2023 study: Same Context Hit vs. Miss difference *M* = 0.007, Variability Context Hit vs. Miss difference *M* = -0.012). The pattern in the current study was also inconsistent across participants, such that 25 had correlations that were numerically in the expected direction whereas 18 did not. Thus, the statistical results indicated no effect: *F*(1, 42) = 0.72, *p* = 0.40, $\eta_{p}^{2}$ = 0.02. We also examined this interaction with the more conservative exclusion criteria previously applied in the Lim et al. study, which produced similar results: *F*(1, 35) = 0.97, *p* = 0.33, $\eta_{p}^{2}$ = 0.03. There was also no three-way interaction between context variability, memory, and hemisphere, *F*(1, 42) = 0.32, *p* = 0.58, $\eta_{p}^{2}$ = 0.007, in the current dataset which was consistent with the Lim et al. findings. Although the key interactions were not significant, hypothesis 1b in our study design table specified a *t*-test to assess any difference between repetitions of subsequently remembered items and subsequently forgotten items in the Variable Context condition. The results of this paired samples *t*-test were consistent with the non-significant interaction term, such that the correlations in these conditions were not significantly different from one another, *t*(42) = 0.35, *p* = 0.72.

**Table 3.** Repeated measures ANOVA results in the hippocampal ROI for the current study and the Lim et al. (2023) study.

		Current study results (*df* = 43)			Lim et al. results (*df* = 29)		
ROI	Effect	*F*	*p*	$\eta_{\text{p}}^{2}$	*F*	*p*	$\eta_{\text{p}}^{2}$
Hipp	Context Variability	2.977	0.092	0.066	9.261	0.005*	0.242
	Memory	0.068	0.795	0.002	0.821	0.372	0.028
	Hemisphere	3.651	0.063	0.080	0.301	0.587	0.010
	Var x Mem	0.717	0.402	0.017	5.465	0.027*	0.159
	Var x Hemi	0.095	0.759	0.002	0.638	0.431	0.022
	Mem x Hemi	1.039	0.314	0.024	0.046	0.831	0.002
	Var x Mem x Hemi	0.315	0.577	0.007	0.820	0.373	0.028

* indicates statistical significance, α = 0.05.

Two findings approached the α of *p* < 0.05 in the hippocampus: a non-significant main effect of hemisphere, *F*(1, 42) = 3.65, *p* = 0.06, $\eta_{p}^{2}$ = 0.08, and non-significant main effect of context variability *F*(1, 42) = 2.98, *p* = 0.09, $\eta_{p}^{2}$ = 0.07. The numerical pattern in the hemispheres indicated that voxel pattern correlations between the first and second presentation of object images were non-significantly larger in the left hippocampus (*M* = 0.039) than in the right hippocampus (*M* = 0.034). There was no effect in this direction in the Lim et al. study (*F*(1, 29) = 0.25, *p* = 0.62, $\eta_{p}^{2}$ = 0.01). The numerical pattern for context variability indicated that the voxel pattern correlations were non-significantly larger in the Same Context condition (*M* = 0.039) than in the Variable Context condition (*M* = 0.033). There was a significant main effect in this direction in the Lim et al. study (*F*(1, 29) = 9.29, *p* = 0.005, $\eta_{p}^{2}$ = 0.24).

The ANOVA of the hippocampal ROI for the current dataset did not approach significance for any other effects. There was no main effect of memory, *F*(1, 42) = 0.07, *p* = 0.80, $\eta_{p}^{2}$ = 0.002. There was no interaction between context variability and hemisphere, *F*(1, 42) = 0.10, *p* = 0.76, $\eta_{p}^{2}$ = 0.002. Both of these non-significant findings were consistent with the Lim et al. (2023) study.

#### 3.2.2 MTL cortex ROI results

The findings for the parahippocampal cortex and perirhinal cortex ROIs were identical to one another in the Lim et al. (2023) study. Both ROIs indicated a significant main effect of hemisphere (right greater than left), a significant main effect of context variability (Same Context greater than Variable Context), a significant interaction between context variability and hemisphere (such that the left hemisphere showed the difference in context variability but the right hemisphere did not) and a significant interaction between memory and hemisphere (such that the right hemisphere showed higher correlations for hits than misses but that the left hemisphere did not). The patterns in parahippocampal cortex and perirhinal cortex differed in the current study although the main effect of context variability was replicated in both ROIs.

#### 3.2.3 Perirhinal cortex ROI

In perirhinal cortex, again using a 2 (context variability) x 2 (memory retrieval) x 2 (hemisphere) repeated measures ANOVA, the current dataset replicated the main effect of context variability found in the Lim et al. (2023) study. Regardless of hemisphere, the perirhinal cortex ROI had larger voxel-level correlations between the first and second presentation of an object image in the Same Context condition (*M* = 0.049) than in the Variable Context condition (*M* = 0.033), *F*(1, 41) = 10.47, *p* = 0.002, $\eta_{p}^{2}$ = 0.20). As indicated in Table 4, this effect was slightly larger than the main effect of context variability seen in the perirhinal cortex ROI in the Lim et al study (*F*(1, 29) = 4.44, *p* = 0.04, $\eta_{p}^{2}$ = 0.13).

**Table 4.** Repeated measures ANOVA results in the perirhinal cortex ROI for the current study and the Lim et al. (2023) study.

		Current study results (*df* = 42)			Lim et al. results (*df* = 29)		
ROI	Effect	*F*	*p*	$\eta_{\text{p}}^{2}$	*F*	*p*	$\eta_{\text{p}}^{2}$
PRc	Context Variability	10.474	0.002*	0.203	4.499	0.043*	0.134
	Memory	5.323	0.026*	0.115	0.282	0.599	0.010
	Hemisphere	0.778	0.383	0.019	19.815	<0.001*	0.406
	Var x Mem	0.902	0.248	0.022	0.148	0.704	0.005
	Var x Hemi	1.556	0.219	0.037	14.316	0.001*	0.331
	Mem x Hemi	1.415	0.421	0.033	7.317	0.011*	0.201
	Var x Mem x Hemi	0.106	0.747	0.003	0.040	0.843	0.001

* indicates statistical significance, α = 0.05.

The Lim et al study’s large main effect of hemisphere in perirhinal cortex, (*F*(1, 29) = 19.66, *p* < 0.001, $\eta_{p}^{2}$ = 0.40), was not found in the current dataset: *F*(1, 41) = 0.078, *p* = 0.38, $\eta_{p}^{2}$ = 0.02). However, the numerical direction of the hemispheric difference from the previous study was maintained (Right *M* = 0.042, Left *M* = 0.039).

A new main effect was found in the current dataset for perirhinal cortex, *F*(1, 41) = 5.32, *p* = 0.03, $\eta_{p}^{2}$ = 0.12), such that trials on which the object images would later be forgotten produced larger voxel correlations (*M* = 0.047) than did trials on which the object images would later be recognized (*M* = 0.035). There was no indication of a main effect of memory in the Lim et al. (2023) study, (*F*(1, 29) = 0.24, *p* = 0.63, $\eta_{p}^{2}$ = 0.01) and the numerical pattern was in the opposite direction to the current finding (Miss *M* = 0.055, Hit *M* = 0.057).

**Fig. 4.**
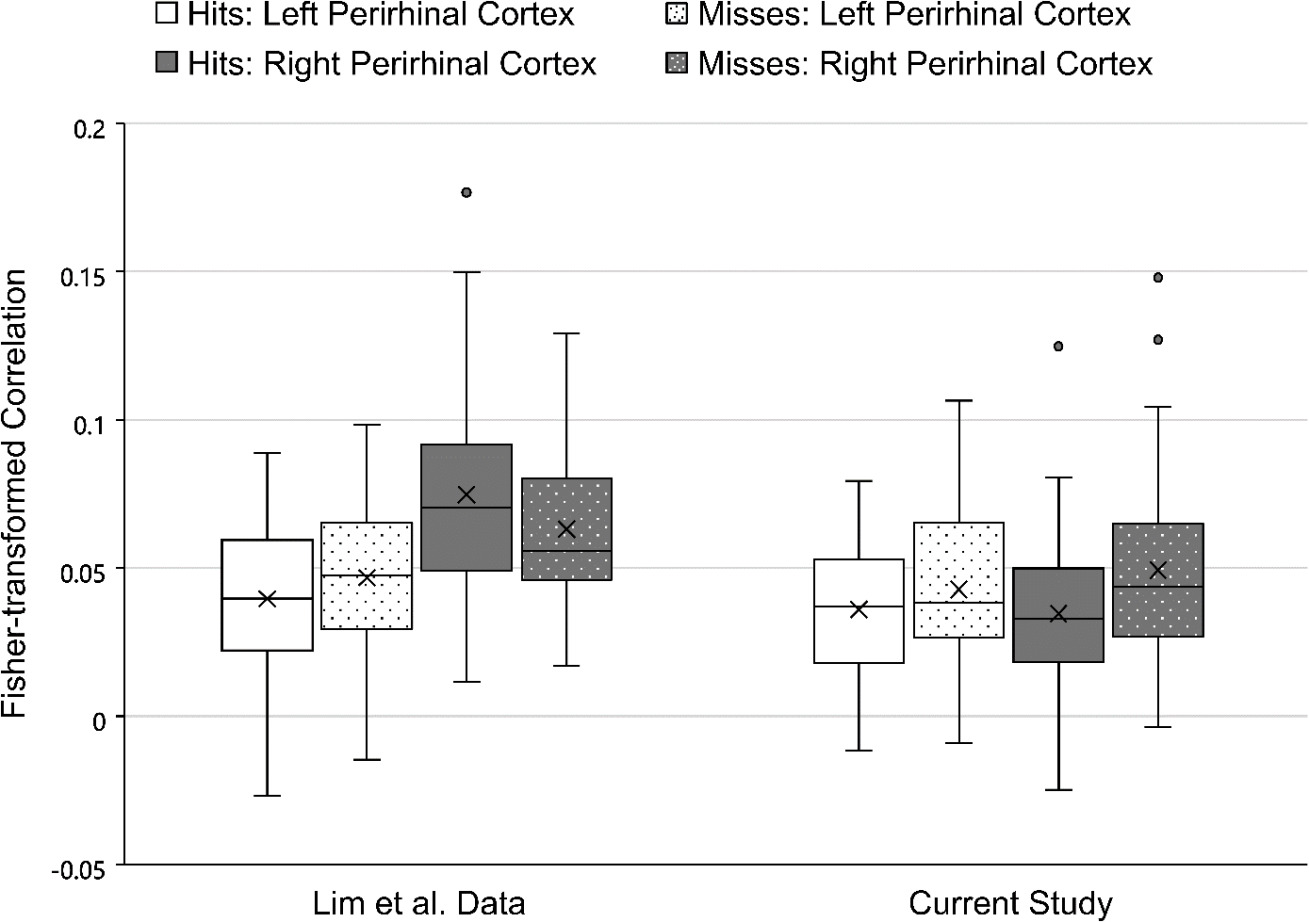
Mean Fisher-transformed correlations for the left and right perirhinal cortex ROIs in the Lim et al. (2023) dataset and the current study, separated according to subsequent memory performance: hits vs. misses. The “X” indicates the mean of each condition and the horizontal line within each box indicates the median of that condition. The center two quartiles of each condition are presented as the box and the lower and upper quartiles are presented as the lower and upper whiskers. Circles indicate individual participants whose correlation value was more than 1.5 times the interquartile range away from the middle two quartiles.

Neither two-way interaction from the Lim et al. (2023) study’s findings in perirhinal cortex was replicated in the current dataset. There was no significant interaction between memory and hemisphere, *F*(1, 41) = 1.42, *p* = 0.24, $\eta_{p}^{2}$ = 0.03 (see Fig. 4), nor was there a significant interaction between context variability and hemisphere, *F*(1, 41) = 1.56, *p* = 0.22, $\eta_{p}^{2}$ = 0.04). Both of these interactions produced moderate effects in the earlier study (memory x hemisphere: *F*(1, 29) = 7.25, *p* = 0.01, $\eta_{p}^{2}$ = 0.20; context variability x hemisphere: *F*(1, 29) = 14.62, *p* < 0.001, $\eta_{p}^{2}$ = 0.34). Instead, in the current dataset, the same effect of higher correlations for Same Context items than Variable Context items was found in both the left (Same Context *M* = 0.046; Variable Context *M* = 0.033) and right (Same Context *M* = 0.051; Variable Context *M* = 0.034) hemispheres. The current dataset also showed a consistent pattern in the left and right hemispheres for memory accuracy (Left Hit vs. Miss *M* = -0.007; Right Hit vs. Miss *M* = -0.016) but it was in the opposite pattern to what was shown only in the right hemisphere in the Lim et al. study (Right Hit vs. Miss *M* = 0.012).

There was no significant interaction between context variability and memory in perirhinal cortex for either the current dataset, *F*(1, 41) = 0.90, *p* = 0.35, $\eta_{p}^{2}$ = 0.02), or the Lim et al. (2023) study, *F*(1, 29) = 1.93, *p* = 0.18, $\eta_{p}^{2}$ = 0.07). Nor was there a significant three-way interaction in perirhinal cortex for either the current dataset, *F*(1, 41) = 0.11, *p* = 0.75, $\eta_{p}^{2}$ = 0.003), or the Lim et al. (2023) study, *F*(1, 29) = 0.04, *p* = 0.84, $\eta_{p}^{2}$ = 0.001).

#### 3.2.4 Parahippocampal cortex ROI

As mentioned above, the pattern found in the current study for parahippocampal cortex differed from the pattern for perirhinal cortex, other than the effect of context variability. A 2 (context variability) x 2 (memory retrieval) x 2 (hemisphere) repeated measures ANOVA for the parahippocampal cortex ROI showed a significant main effect of context variability: *F*(1, 42) = 13.19, *p* < 0.001, $\eta_{p}^{2}$ = 0.24). As in perirhinal cortex, this effect was driven by larger voxel-level correlations between the first and second presentation of an object image in the Same Context condition (*M* = 0.067) than in the Variable Context condition (*M* = 0.052). As indicated in Table 5, this effect was slightly larger than the main effect of context variability seen in the Lim et al study (*F*(1, 29) = 7.43, *p* = 0.01, $\eta_{p}^{2}$ = 0.20).

**Table 5.** Repeated measures ANOVA results in the parahippocampal cortex ROI for the current study and the Lim et al. (2023) study.

		Current study results (*df* = 43)			Lim et al. results (*df* = 29)		
ROI	Effect	*F*	*p*	$\eta_{\text{p}}^{2}$	*F*	*p*	$\eta_{\text{p}}^{2}$
PHc	Context Variability	13.193	<0.001*	0.239	7.982	0.008*	0.216
	Memory	0.220	0.641	0.005	2.541	0.122	0.081
	Hemisphere	0.516	0.477	0.012	10.056	0.004*	0.257
	Var x Mem	1.011	0.320	0.024	0.097	0.757	0.003
	Var x Hemi	2.377	0.131	0.054	9.812	0.004*	0.253
	Mem x Hemi	1.208	0.278	0.028	6.464	0.017*	0.182
	Var x Mem x Hemi	0.052	0.821	0.001	0.709	0.407	0.024

* indicates statistical significance, α = 0.05.

**Fig. 5.**
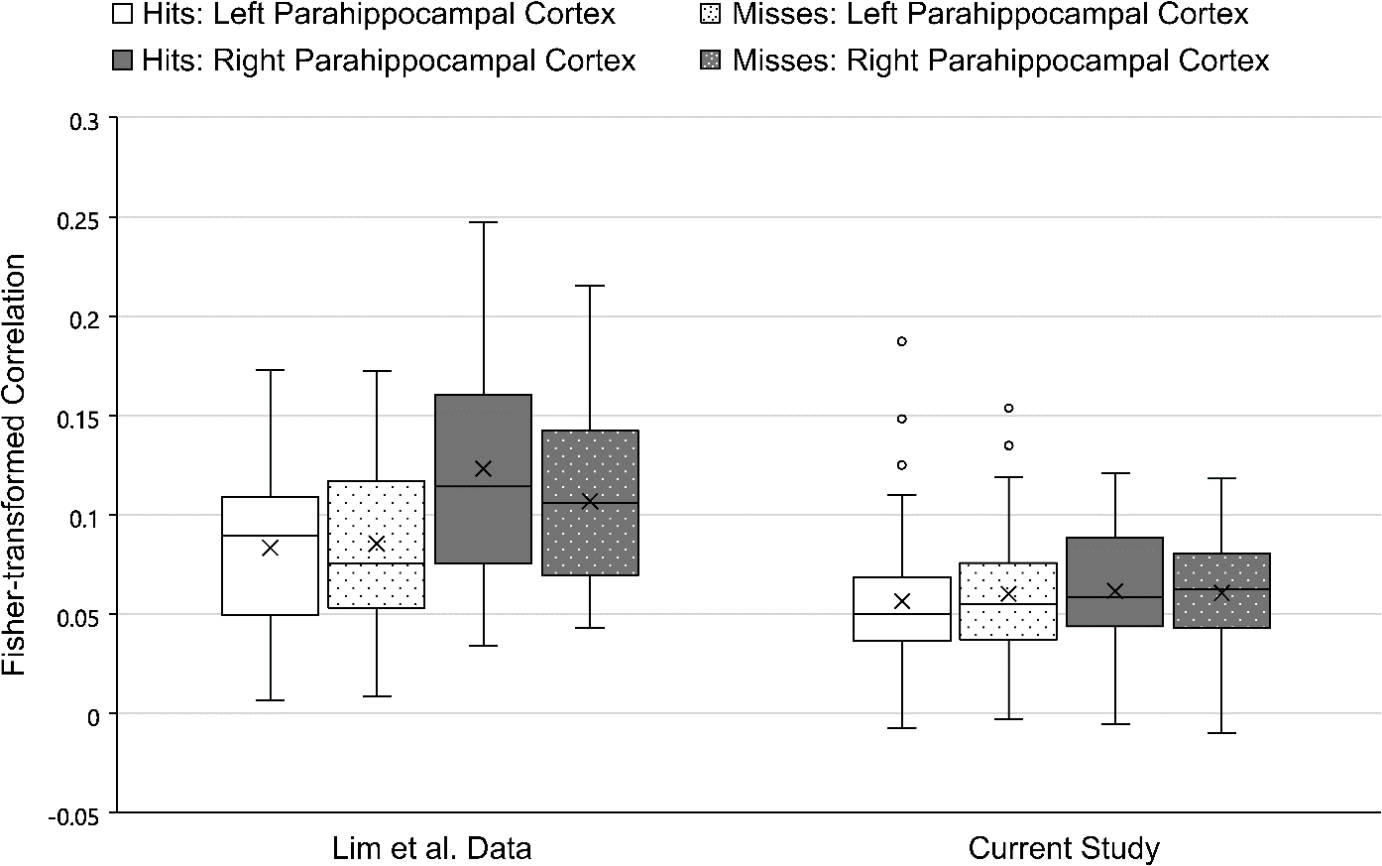
Mean Fisher-transformed correlations for the left and right parahippocampal cortex ROI in the Lim et al. (2023) dataset and the current study, separated according to subsequent memory performance: hits vs. misses. The “X” indicates the mean of each condition and the horizontal line within each box indicates the median of that condition. The center two quartiles of each condition are presented as the box and the lower and upper quartiles are presented as the lower and upper whiskers. Circles indicate individual participants whose correlation value was more than 1.5 times the interquartile range away from the middle two quartiles.

The Lim et al study’s main effect of hemisphere in parahippocampal cortex, (*F*(1, 29) = 10.22, *p* = 0.003, $\eta_{p}^{2}$ = 0.26), was not found in the current dataset: *F*(1, 42) = 0.52, *p* = 0.48, $\eta_{p}^{2}$ = 0.01). However, the numerical direction of the hemispheric difference from the previous study was maintained (Right *M* = 0.061, Left *M* = 0.058). There was also no significant interaction between memory and hemisphere in the current study, *F*(1, 42) = 1.21, *p* = 0.28, $\eta_{p}^{2}$ = 0.03), despite a moderate effect in the earlier study, *F*(1, 29) = 6.72, *p* = 0.02, $\eta_{p}^{2}$ = 0.19. Instead, as shown in Figure 5, both hemispheres showed very little difference in the correlations between later remembered items and later forgotten items (Left Hit vs. Miss *M* = 0.003; Right Hit vs. Miss *M* = -0.005). In contrast, the Lim et al. study showed a significant interaction driven by a moderate difference in the right hemisphere only (Left Hit vs. Miss *M* = -0.002; Right Hit vs. Miss *M* = 0.016).

Finally, there was no significant interaction between context variability and hemisphere, *F*(1, 42) = 2.38, *p* = 0.13, $\eta_{p}^{2}$ = 0.05), despite a moderate effect in the earlier study: *F*(1, 29) = 10.05, *p* = 0.004, $\eta_{p}^{2}$ = 0.26). Although the Lim et al. study found that Same Context correlations were higher than Variable context correlations only in the left hemisphere, the current dataset identified the same effect in both the left and right hemispheres (Left Same Context *M* = 0.064; Left Variable Context *M* = 0.053; Right Same Context *M* = 0.071; Variable Context *M* = 0.052).

Consistent with the Lim et al. (2023) study, there was no main effect of memory in the parahippocampal cortex in the current study: *F*(1, 42) = 0.22, *p* = 0.64, $\eta_{p}^{2}$ = 0.005). There was no interaction between context variability and memory in parahippocampal cortex for either the current dataset, *F*(1, 42) = 1.01, *p* = 0.32, $\eta_{p}^{2}$ = 0.02), or the Lim et al. (2023) study, *F*(1, 29) = 0.10, *p* = 0.76, $\eta_{p}^{2}$ = 0.003). Nor was there a significant three-way interaction in perirhinal cortex for either the current dataset, *F*(1, 42) = 0.52, *p* = 0.82, $\eta_{p}^{2}$ = 0.001), or the Lim et al. (2023) study, *F*(1, 29) = 0.65, *p* = 0.43, $\eta_{p}^{2}$ = 0.02.

#### 3.2.5 Lateral occipital cortex ROI results

The Lateral Occipital Cortex (LOC) region in the current study replicated one main effect from the Lim et al. (2023) study. A 2 (context variability) x 2 (memory retrieval) x 2 (hemisphere) repeated measures ANOVA for the LOC ROI also showed a large significant main effect of context variability: *F*(1, 42) = 30.98, *p* < 0.001, $\eta_{p}^{2}$ = 0.42). This was comparable with the large main effect of context variability found in the Lim et al. study: *F*(1, 29) = 55.13, *p* < 0.001, $\eta_{p}^{2}$ = 0.66) as indicated in Table 6. Both effects reflected larger voxel pattern correlations in LOC for the Same Context condition (current study *M* = 0.112; Lim et al. study *M* = 0.213) than for the Variable Context condition (current study *M* = 0.093; Lim et al. study *M* = 0.182).

**Table 6.** Repeated measures ANOVA results in the lateral occipital cortex ROI for the current study and the Lim et al. (2023) study.

		Current study results (*df* = 43)			Lim et al. results (*df* = 29)		
ROI	Effect	*F*	*p*	$\eta_{\text{p}}^{2}$	*F*	*p*	$\eta_{\text{p}}^{2}$
LOC	Context Variability	30.976	<0.001*	0.424	58.252	<0.001*	0.668
	Memory	0.957	0.334	0.022	10.367	0.003*	0.263
	Hemisphere	1.805	0.186	0.041	33.184	<0.001*	0.534
	Var x Mem	4.373	0.043*	0.094	1.992	0.169	0.064
	Var x Hemi	14.776	<0.001*	0.260	2.142	0.154	0.069
	Mem x Hemi	0.024	0.878	0.001	1.806	0.189	0.059
	Var x Mem x Hemi	0.061	0.807	0.001	0.440	0.512	0.015

* indicates statistical significance, α = 0.05.

However, the current findings did not indicate either of the two additional main effects found for LOC in the Lim et al. (2023) study. There was no significant main effect of memory, *F*(1, 42) = 0.96, *p* = 0.33, $\eta_{p}^{2}$ = 0.02 in the current dataset, despite a moderate effect in the earlier study: *F*(1, 29) = 10.00, *p* = 0.004, $\eta_{p}^{2}$ = 0.26. There was no significant main effect of hemisphere, *F*(1, 42) = 1.81, *p* = 0.19, $\eta_{p}^{2}$ = 0.04, despite a large effect in the earlier study: *F*(1, 29) = 29.52, *p* < 0.001, $\eta_{p}^{2}$ = 0.50.

**Fig. 6.**
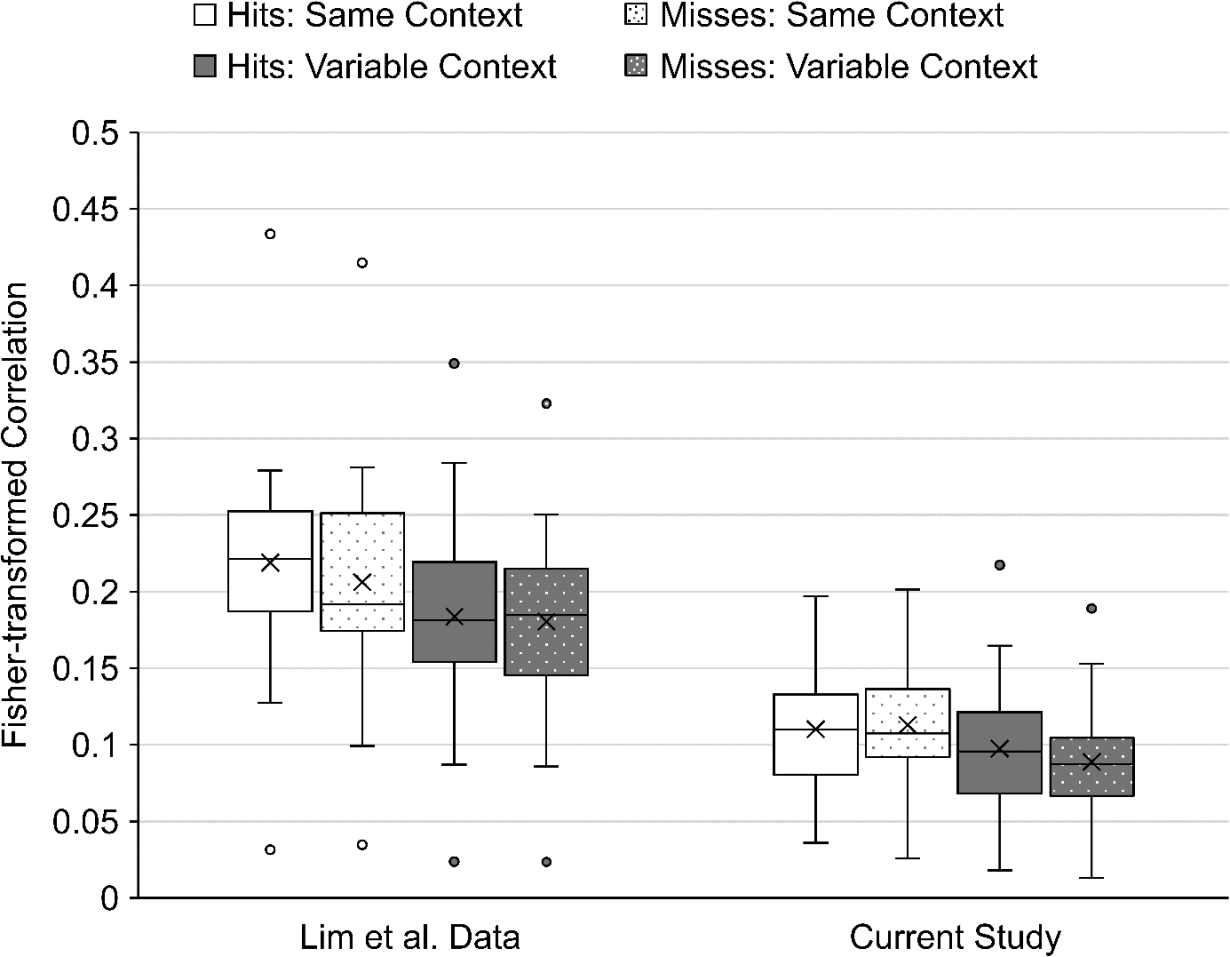
Mean Fisher-transformed correlations for the bilateral LOC ROI in the Lim et al. (2023) dataset and the current study, highlighting the significant interaction in the current study between context variability (Same Context vs. Variable Context) and subsequent memory. The “X” indicates the mean of each condition and the horizontal line within each box indicates the median of that condition. The center two quartiles of each condition are presented as the box and the lower and upper quartiles are presented as the lower and upper whiskers. Circles indicate individual participants whose correlation value was more than 1.5 times the interquartile range away from the middle two quartiles.

The LOC region did indicate a new context variability by memory interaction in the current dataset: *F*(1, 42) = 4.37, *p* = 0.04, $\eta_{p}^{2}$ = 0.09. This effect was not significant in the Lim et al. (2023) study: *F*(1, 29) = 1.99, *p* = 0.17, $\eta_{p}^{2}$ = 0.06. As shown in Figure 6, the pattern of the interaction in LOC differed from the context variability by memory interaction that was found in the hippocampus in the Lim et al. study. In the current dataset, LOC produced larger voxel pattern correlations in the Variable Context condition when those items would later be remembered (*M* = 0.042) than when those items would later be forgotten (*M* = 0.038), *t*(42) = 2.27, *p* = 0.03. There was no difference between correlations in the Same Context condition for remembered (*M* = 0.040) vs. forgotten items (*M* = 0.040), *p* = 0.48.

The current dataset also indicated a new interaction in LOC between context variability and hemisphere: *F*(1, 42) = 14.78, *p* < 0.001, $\eta_{p}^{2}$ = 0.26. This two-way interaction was not significant in the Lim et al. (2023) study: *F*(1, 29) = 2.14, *p* = 0.15, $\eta_{p}^{2}$ = 0.07.

There was no interaction between memory and hemisphere in LOC in the current dataset: *F*(1, 42) = 0.02, *p* = 0.88, $\eta_{p}^{2}$ = 0.001. This was consistent with the Lim et al. (2023) study: *F*(1, 29) = 1.81, *p* = 0.19, $\eta_{p}^{2}$ = 0.06. There was no three-way interaction between the factors of context variability, memory, and hemisphere in LOC in the current dataset: *F*(1, 42) = 0.06, *p* = 0.81, $\eta_{p}^{2}$ = 0.001. This was consistent with the Lim et al. study: *F*(1, 29) = 0.44, *p* = 0.51, $\eta_{p}^{2}$ = 0.02.

### 3.3 Lim et al. re-analysis with the current (more liberal) inclusion criteria

As described in section 2.1 above, mid-way through data collection but prior to hypothesis testing for the current dataset, we deviated from the protocol’s pre-registered inclusion criteria with approval from the registered report’s reviewers. The altered criteria set a minimum *d′* at 0.50 (rather than 0.75) and minimum number of usable trials at 19 per condition (rather than 20 trials). Given that these new criteria did not precisely match the inclusion criteria for the Lim et al. (2023) study, we planned at that point to add a secondary analysis of the Lim et al. data with the same alterations to the inclusion criteria. Upon review of the Lim et al. participants, we found that two previously removed participants met the new inclusion criteria, for a total N of 32 in this re-analysis. The two added participants were both previously excluded due to the *d′* criterion, with one having an average *d′* of 0.70 and the other having an average *d′* of 0.68. With these additional participants, the behavioral findings were unchanged: the Same Context condition produced fewer hits (*M* = 0.46) than did the Variable Context condition (*M* = 0.54, *t*(31) = 8.09, *p* < 0.001).

The re-analysis of the RSA correlations indicated some meaningful differences with these two participants added to the set, but only in the hippocampal and LOC ROIs. First, the primary interaction of interest in the hippocampus, between context variability and memory accuracy, no longer met the 0.05 alpha level for significance: *F*(1, 31) = 3.45, *p* = 0.07, $\eta_{p}^{2}$ = 0.10. The significant main effect of context and the lack of other main effects or interactions were unchanged in this re-analysis. In LOC, most findings remained the same (significant main effects of context, accuracy, and hemisphere). However, the interaction between context variability and memory accuracy now approached, but did not reach, significance: *F*(1, 31) = 3.15, *p* = 0.09, $\eta_{p}^{2}$ = 0.09. This change was particularly interesting because the current dataset identified this interaction as being significant.

## 4 Discussion

The goal of this study was to assess the replicability of RSA findings that indicate whether voxel pattern similarity or dissimilarity across repeated events is associated with subsequent memory retrieval. Our previous study, Lim et al. (2023), proposed that inconsistencies in the existing literature on this topic may have been driven by differences in cognitive goals influencing whether pattern similarity or dissimilarity predicts memory success. However, an alternative explanation was that subsequent memory RSA effects suffer from sensitivity to minor methodological differences and, therefore, may fail to replicate. In order to test this alternative hypothesis, the current study was a registered replication attempt using an identical behavioral procedure and fMRI analysis but with several minor variations to the fMRI methods: voxel size, slice orientation, and intertrial interval. We predicted that we would either replicate the hippocampal two-way interaction between context variability and subsequent memory retrieval or find a three-way interaction that also included hemisphere as a relevant factor. This prediction was not supported. Neither the context variability x memory interaction or the three-way interaction including hemisphere approached significance. Although the numerical pattern of smaller correlations for Variable Context Hits than Misses was maintained, the differences between the means were reduced from a difference of -0.012 (Variable Context Hit vs. Miss) in the Lim et al. dataset to a difference of -0.002 in the current dataset and the pattern did not appear in the individual correlation data for 18 out of 43 participants. Thus, we conclude that we have failed to replicate our findings. And, as stated in our pre-registered hypotheses, we must “conclude that methodological inconsistency or other as-yet-unknown factors drive variability in hippocampal RSA findings in the literature.”

It should be noted that our re-analysis of the Lim et al. (2023) dataset using slightly more liberal response criteria (see Section 2.3) did not produce a significant interaction between context variability and subsequent memory in the hippocampus (*p* = 0.07). This re-analysis included just two additional participants, but that change was sufficient to change the outcome. This re-analysis in itself suggests that the Lim et al. hippocampal finding was not robust. However, we do not think this affects our conclusion that RSA findings related to subsequent memory are sensitive to minor methodological differences. Even if the hippocampal finding is ignored, none of the other effects related to subsequent memory in the other three ROIs were replicated. Notably, there was a significant main effect of memory in perirhinal cortex in the current dataset (larger correlations for forgotten items than remembered items) that contrasted with the opposite pattern found in a significant memory x hemisphere interaction in perirhinal cortex in the Lim et al. study (larger correlations for remembered items than for forgotten items in the right hemisphere only). In parahippocampal cortex, the significant memory x hemisphere interaction from the Lim et al. study (*p* = 0.02, $\eta_{p}^{2}$ = 0.19) was not replicated in the current dataset (*p* = 0.28, $\eta_{p}^{2}$ = 0.03). Finally, the main effect of memory in LOC from the Lim et al. study (*p* = 0.004, $\eta_{p}^{2}$ = 0.26), such that later remembered items produced larger correlations than later forgotten items, was not found in the current study. Instead, in the current study, we found an interaction between context variability and memory (*p* = 0.04, $\eta_{p}^{2}$ = 0.09) such that the increase in correlations for remembered items vs. forgotten items only occurred within the Variable Context condition, whereas there was no difference in correlation size due to future memory status in the Same Context condition. Thus, even if the hippocampal interaction of context variability x subsequent memory is discounted as a target for replication due to its unreliability in the Lim et al. re-analysis, there is evidence from our other three ROIs that subsequent memory RSA effects are sensitive to minor methodological variance.

Our initial reaction to this replication failure was to consider whether some defect in the functional scanning sequence prevented us from finding meaningful data in the current study. However, that conclusion does not appear to be supported by the overall range of findings. Although many findings from the Lim et al. (2023) study were not significant in the current dataset, there were other effects that were statistically significant in the current dataset but did not appear in the Lim et al. study. Thus, the current scanning sequence did find measurable correlations that changed with the behavioral manipulation. Also, of the three statistical effects that replicated, two had larger effect sizes in the current study (parahippocampal cortex main effect of context variability: current study $\eta_{p}^{2}$ = 0.24 vs. Lim et al. $\eta_{p}^{2}$ = 0.20; perirhinal cortex main effect of context variability: current study $\eta_{p}^{2}$ = 0.20 vs. Lim et al. $\eta_{p}^{2}$ = 0.13), whereas one had a larger effect size in the Lim et al. study (LOC main effect of context variability: current study $\eta_{p}^{2}$ = 0.42 vs. Lim et al. $\eta_{p}^{2}$ = 0.66). Thus, we do not think the modified scanning sequence in the current study was simply a poorer measure of BOLD signal changes due to cognition than the sequence in the Lim et al. study.

Given the overall lack of replicability across the majority of the RSA findings between the Lim et al. (2023) study and the current dataset, we are reluctant to interpret the statistically significant findings in the new dataset. At best, it might be appropriate to interpret those findings that were significant in both studies. The replicated effects include main effects of context variability in the perirhinal and parahippocampal cortices and in LOC. The effect sizes were slightly larger in the current study for perirhinal and parahippocampal cortices but were slightly larger in the Lim et al. study for LOC. In all three ROIs, the effect indicated higher correlations for Same Context trial repetitions than for Variable Context trial repetitions. Although this may mean that these effects are reliable RSA findings, it is important to note that they do not include effects driven by subsequent memory retrieval. In addition, all three effects can be explained purely based on minor visual differences between the stimulus presentations in the Same Context condition and the Variable Context condition. That is, the on-screen encoding question presented at the top of the screen was identical for the first and second viewings of the object image in the Same Context condition (either “Is this item useful on a deserted island?” or “Could you carry this item a long distance?”). In the Variable Context condition, the object image and other screen characteristics were identical, but the eight words at the top of the screen were different between the first and second presentations of each trial. Therefore, we conclude that our RSA findings were reliable with respect to visual similarities and differences during memory encoding but were not reliable with respect to the likelihood of future memorability.

Two of the methodological changes made in the current study: slice orientation and voxel size, were intentionally chosen because they have not previously been identified as critical factors in RSA approaches. Although individual researchers may prefer an axial, sagittal, or coronal slice orientation, these selections are often based on signal dropout near air–tissue boundaries or the degree of head coverage provided. We are not aware of any consistent evidence that one orientation is preferable to the others when signal dropout is not an issue. For example, axial slices that are anatomically aligned sometimes produce better data than coronal slices (Gustard et al., 2001) or reduce dropout in the temporal lobe (Theysohn et al., 2013), but in other circumstances oblique pseudo-coronal slices are identified as preferable (in the amygdala: Chen et al., 2003) or axial slices produce more artifact than sagittal or coronal slices (Ojemann et al., 1997). We did not observe signal dropout in the hippocampus in any of our participants, but slice orientation may have affected perirhinal cortex coverage. In the current study, we had one participant with no detectible signal in perirhinal cortex (using axial slices), whereas this did not occur in the Lim et al. (2023) dataset (using sagittal slices, which were chosen to reduce dropout).

Although there is no single ideal voxel size for RSA identified in the literature, there is agreement that smaller voxels reduce signal-to-noise ratio but that larger voxels increase the degree of partial volume effects (Du et al., 2014). Partial volume effects occur when individual voxels include qualitatively different tissue (e.g., including both white and gray matter). This may be particularly relevant in the hippocampus both because it is surrounded by white matter and because the structure is composed of layers that may have different functions (Palomero-Gallagher et al., 2020). Studies of partial volume effects that also consider contrast to noise have identified an optimum voxel size of 1.5 mm^3^ (Hyde et al., 2001), or 2 mm^3^ (Weibull et al., 2008). It has been proposed that high-field MRI (7T) may be ideal for RSA because it increases BOLD contrast and allows for smaller voxels with reduced partial volume effects (Formisano & Kriegeskorte, 2012) but high-field studies remain relatively rare. If smaller voxels are preferable for RSA, then we might conclude that the current dataset (using voxels of 2.7 mm^3^) provides more accurate findings than the Lim et al (2023) study (using voxels of 3.0 mm^3^). However, it should be noted that the voxel sizes in both datasets are well within the range of typical voxel sizes in the existing RSA literature. Additional consideration of voxel sizes when comparing prior studies and in designing future studies may be important for identifying the replicability of RSA effects.

The third methodological change made in the current study, ITI, is known to be important for RSA outcomes (Dimsdale-Zucker & Ranganath, 2018), but was altered in a way that remained within the range of recommended values for RSA. ITI is a key factor in the statistical modeling that attempts to identify the BOLD signal associated with a cognitive event. Prior methodological studies evaluating ranges of ITI lengths for fMRI individual trial modeling, using LSS models, agreed that a jittered ITI averaging 4 to 6 s produced optimal results with no benefit to increasing the ITI length (Abdulrahman & Henson, 2016; Mumford et al., 2014). Therefore, the average ITI of 5 s (with each trial ITI at 4 s, 6 s, or 8 s) in the current study and the average ITI of 6 s (with each trial ITI at 4 s, 6 s, 8 s, or 10 s) in the Lim et al. (2023) dataset were both optimal. At least one methodological paper has argued that longer ITI lengths that are not jittered are preferable for fMRI studies using individual trial modeling (Zeithamova et al., 2017); however, this difference was not evident when LSS modeling was used. If that study’s overall argument is correct, then we might conclude that the Lim et al. (2023) study provides more accurate findings than the current dataset, which conflicts with the possible implications for voxel size raised above. Again, we suggest that additional consideration of ITI lengths may be needed when comparing RSA effects.

In summary, we conclude that the current study supports the claim that variability in the fMRI RSA literature for subsequent memory effects may be driven by methodological inconsistencies rather than meaningful differences in representational patterns. Of the methodological changes made in this replication study, it is not clear which (voxel size, slice orientation, or intertrial interval) had the largest influence on the results. We propose that additional replication tests of existing studies are needed to verify or refute this conclusion. In addition, modeling or methodological studies may be helpful to further refine best practices for imaging parameters and design decisions when using RSA techniques.

## Data Availability

All raw data, custom scripts, RSA output, and statistical output are available at: https://doi.org/10.17605/OSF.IO/ZUYNA

## Footnotes

1 The original Stage 1 protocol proposed a voxel size of 2.4 mm isotropic and a field of view of 156 mm. Prior to data collection, we requested and were approved by the recommenders to revise this protocol to allow a larger field of view (192 mm) by increasing the voxel size to 2.7 mm isotropic.
